# Supplementary material for: Association between SNPs in microRNAs and microRNAs-Machinery Genes with Susceptibility of Leprosy in the Amazon Population
Source: Int J Mol Sci. 2022 Sep 13;23(18):10628. doi: 10.3390/ijms231810628 (PMC9503809; doi:10.3390/ijms231810628)
Supplement: Supplementary file 1 [file ijms-23-10628-s001.zip › ijms-1869802-supplementary.pdf]

Table S1. Genotypic distribution of the investigated polymorphisms for leprosy patients in comparison to control group to markers associated with susceptibility to leprosy.

Gene	Genotype	Case (%)	Control (%)	*p*-value	Model	OR (95%CI)
miR300	rs12894467	111	68			
	TT	44 (39.6)	30 (44.1)	0.76	TT vs TC + CC	1.10(0.49-2.45)
	CT	45 (40.5)	26 (38.2)			
	CC	22 (19.8)	12 (17.6)	0.82	TT + TC vs CC	1.10(0.49-2.45)
miR423	rs6505162	93	59			
	AA	27 (29.0)	15 (25.4)	0.71	AA vs AC + CC	1.15(0.40-1.86)
	AC	47 (50.5)	32 (54.2)			
	CC	19 (20.4)	12 (20.3)	0.87	AA + AC vs CC	1.07(0.45-2.53)
mir604	rs2368392	83	54			
	AA	42 (50.6)	24 (44.4)	0.48	AA vs AG + GG	0.77(0.36-1.61)
	AG	40 (48.2)	29 (53.7)			
	GG	1 (1.2)	1 (1.9)	0.69	AA + AG vs GG	0.57(0.03-1.67)
pre-miR938	rs2505901	109	68			
	TT	26 (23.9)	27 (39.7)	**<0.01**	TT vs TC + CC	**0.40(0.20-0.81)**
	CT	53 (48.6)	30 (44.1)			
	CC	30 (27.5)	11 (16.2)	**0.03**	TT + CT vs CC	**0.41(0.18-0.95)**

miR100	rs1834306	93	58			
	AA	44 (47.3)	25 (43.1)	0.60	AA vs AG + GG	0.83(0.42-1.66)
	AG	36 (38.7)	26 (44.8)			
	GG	13 (14.0)	7 (12.1)	0.99	AA + AG vs GG	1.01(0.36-2.77)
miR219A1	rs213210	140	70			
	AA	91 (81.2)	58 (84.1)	0.60	AA vs AG + GG	1.25(0.54-2.86)
	AG	21 (18.8)	9 (13.0)			
	GG	0 (0.0)	2 (2.9)	0.14	AA + AG vs GG	0.00 (0.00)
DROSHA	rs639174	101	64			
	CC	32 (31.7)	30 (46.9)	**0.02**	CC vs CT + TT	**0.45(0.23-0.89)**
	CT	51 (50.5)	28 (43.8)			
	TT	18 (17.8)	6 (9.4)	0.16	CC + CT vs TT	2.00(0.73-5.51)
miR453	rs56103835	109	69			
	TT	60 (55.0)	32 (46.4)	0.42	TT vs CT + CC	0.77(0.41-1.45)
	CT	38 (34.9)	30 (43.5)			
	CC	11 (10.1)	7 (10.1)	0.96	TT + CT vs CC	0.98(0.35-2.72)
miR196A2	rs11614913	101	66			
	CC	61 (60.4)	31 (47.0)	0.08	CC vs CT + TT	0.56(0.29-1.08)

	CT	31 (30.7)	30 (45.5)			
	TT	9 (8.9)	5 (7.6)	0.64	CC + CT vs TT	1.32(0.40-4.35)
AGO	rs636832	102	66			
	GG	46 (45.1)	39 (59.1)	**0.01**	GG vs GA + AA	**0.45(0.23-0.88)**
	GA	43 (42.2)	21 (31.8)			
	AA	13 (12.7)	6 (9.1)	0.42	GG + GA vs AA	0.66(0.23-1.86)
pri-let-7a1	rs10739971	30	37			
	GG	15 (50.0)	6 (22.2)	**0.02**	GG vs GA + AA	**4.66(1.17-18.66)**
	GA	12 (40.0)	14 (51.9)			
	AA	3 (10.0)	7 (25.9)	**0.04**	GG + GA vs AA	**5.09(0.95-7.45)**
miR146A	rs2910164	108	69			
	GG	47 (43.5)	29 (42.0)	0.67	GG vs GC + CC	0.87(0.46-1.65)
	GC	54 (50.0)	35 (50.7)			
	CC	7 (6.5)	5 (7.2)	0.66	GG + GC vs CC	1.31(0.22-2.59)
miR570	rs4143815	111	69			
	GG	62 (55.9)	46 (66.7)	0.73	GG vs GC + CC	1.41(0.73-2.73)
	GC	38 (34.2)	19 (27.5)			
	CC	11 (9.9)	4 (5.8)	0.49	GG + GC vs CC	1.64(0.49-5.50)
miR200B	rs9660710	103	67			

	CC	79 (76.7)	55 (82.1)	0.62	CC vs CA + AA	1.22 (0.54-2.79)
	CA	22 (21.4)	12 (17.9)			
	AA	2 (1.9)	0 (0.0)	0.14	CC + CA vs AA	1.00 (0.00)
miR26-A1	rs7372209	98	62			
	CC	44 (44.9)	33 (53.2)	0.32	CC vs CT + TT	1.40(0.72-2.72)
	CT	43 (43.9)	23 (37.1)			
	TT	11 (11.2)	6 (9.7)	0.41	CC + CT vs TT	1.59(0.51-4.94)
miR200C	rs12904	111	69			
	GG	36 (32.4)	11 (15.9)	**0.01**	GG vs AG + AA	**2.77(1.25-6.11)**
	AG	53 (47.7)	45 (65.2)			
	AA	22 (19.8)	13 (18.8)	0.92	GG + AG vs AA	1.04(0.43-2.15)
DROSHA	rs10035440	110	69			
	TT	80 (72.7)	40 (58.0)	**0.03**	TT vs CT + CC	**2.04(1.03-4.02)**
	CT	29 (26.4)	27 (39.1)			
	CC	1 (0.9)	2 (2.9)	0.27	TT + CT vs CC	3.82(0.32-3.10)
miR4513	rs2168518	112	69			
	GG	51 (45.5)	43 (62.3)	0.19	GG vs GA + AA	1.56(0.80-3.04)
	GA	53 (47.3)	20 (29.0)			
	AA	8 (7.1)	6 (8.7)	0.38	GG + AG vs AA	1.68(0.53-5.34)

miR219-1	rs107822	95	64			
	CC	58 (61.1)	37 (57.8)	0.73	CC vs CT + TT	0.89(0.46-1.74)
	CT	28 (29.5)	23 (35.9)			
	TT	9 (9.5)	4 (6.2)	0.52	CC + CT vs TT	1.49(0.42-1.55)
miR2053	rs10505168	105	66			
	TT	45 (42.9)	25 (37.9)	0.66	TT vs TC + CC	0.87(0.45-1.67)
	TC	45 (42.9)	29 (43.9)			
	CC	15 (14.3)	12 (18.2)	0.63	TT + CT vs CC	0.81(0.34-1.90)
miR499	rs3746444	109	69			
	GG	79 (72.5)	53 (76.8)	0.62	GG vs GA + AA	1.20(0.57-2.50)
	GA	26 (23.9)	14 (20.3)			
	AA	4 (3.7)	2 (2.9)	0.61	GG + GA vs AA	1.62(0.24-10.69)

*Sample number; *P*-value, OR and 95%CI were obtained with logistic regression adjusted for genetic ancestry.

Table S2. Genotypic distribution of the investigated polymorphisms for leprosy patients grouped in clinical form PB in comparison to the control group.

Gene	Genotype	PB (%)	Control (%)	*p*-value	Model	OR (95%CI)
miR300	rs12894467	55	68			
	TT	18 (32.7)	30 (44.1)	0.22	TT vs TC + CC	1.63(0.74-3.59)
	TC	24 (43.6)	26 (38.2)			
	CC	13 (23.6)	12 (17.6)	0.48	TT + TC vs CC	1.39(0.55-3.50)
	Alelo T	0.44	0.53			
	Alelo C	0.56	0.46			
miR423	rs6505162	49	59			
	AA	12 (24.5)	15 (25.4)	0.79	AA vs AC + CC	1.13(0.45-2.83)
	AC	26 (53.1)	32 (54.2)			
	CC	11 (22.4)	12 (20.3)	0.42	AA + AC vs CC	1.14(0.42-3.08)
	Alelo A	0.38	0.39			
	Alelo C	0.62	0.61			
mir604	rs2368392	43	54			
	AA	21 (48.8)	24 (44.4)	0.64	AA vs AG + GG	0.81(0.33-2.00)
	AG	22 (51.2)	29 (53.7)			
	GG	0 (0.0)	1 (1.9)	0.23	AA + AG vs GG	0.00(0.00)
	Alelo A	0.74	0.71			

	Alelo G	0.26	0.29			
pre-miR938	rs2505901	53	68			
	TT	11 (20.8)	27 (39.7)	**<0.01**	TT vs TC + CC	**0.31(0.12-0.75)**
	TC	25 (47.2)	30 (44.1)			
	CC	17 (32.1)	11 (16.2)	**0.01**	TT + CT vs CC	**0.31(0.12-0.84)**
	Alelo T	0.44	0.62			
	Alelo C	0.56	0.38			
miR100	rs1834306	48	58			
	AA	24 (50.0)	25 (43.1)	0.42	AA vs AG + GG	0.72(0.32-1.63)
	AG	17 (35.4)	26 (44.8)			
	GG	7 (14.6)	7 (12.1)	0.77	AA + AG vs GG	1.19(0.37-3.87)
	Alelo A	0.68	0.65			
	Alelo G	0.32	0.35			
miR219A1	rs213210	55	69			
	AA	42 (76.4)	58 (84.1)	0.38	AA + AG vs GG	1.52(0.59-3.94)
	AG	13 (23.6)	9 (13.0)			
	GG	0 (0.00)	2 (2.9)	0.06	AA vs AG + GG	0.00 (0.00)
	Alelo A	0.88	0.90			
	Alelo G	0.12	0.10			

DROSHA	rs639174	50	64			
	CC	16 (32.0)	30 (46.9)	0.08	CC vs CT + TT	0.50(0.22-1.12)
	CT	27 (54.0)	28 (43.8)			
	TT	7 (14.0)	6 (9.4)	0.60	CC + CT vs TT	0.72(0.21-2.31)
	Alelo C	0.59	0.69			
	Alelo T	0.41	0.31			
miR453	rs56103835	54	69			
	TT	30 (55.6)	32 (46.4)	0.52	TT vs CT + CC	0.78(0.36-1.67)
	CT	20 (37.0)	30 (43.5)			
	CC	4 (7.4)	7 (10.1)	0.53	TT + CT vs CC	0.66(0.17-2.53)
	Alelo T	0.74	0.68			
	Alelo C	0.26	0.32			
miR196A2	rs11614913	52	66			
	CC	32 (61.5)	31 (47.0)	0.12	CC vs CT + TT	0.53(0.24-1.19)
	CT	14 (26.9)	30 (45.5)			
	TT	6 (11.5)	5 (7.6)	0.24	CC + CT vs TT	2.29(0.56-9.30)
	Alelo C	0.75	0.70			
	Alelo T	0.25	0.30			
AGO	rs636832	52	66			

	GG	24 (46.2)	39 (59.1)	0.11	GG vs GA + AA	0.53(0.24-1.16)
	GA	20 (38.5)	21 (31.8)			
	AA	8 (15.4)	6 (9.1)	0.28	GG + GA vs AA	0.53(0.16-1.73)
	Alelo G	0.65	0.75			
	Alelo A	0.35	0.25			
pri-let-7a1	rs10739971	13	27			
	GG	9 (69.2)	6 (22.2)	**<0.01**	GG vs GA + AA	**13.3(1.82-90.0)**
	GA	3 (23.1)	14 (51.9)			
	AA	1 (7.7)	7 (25.9)	0.09	GG + GA vs AA	6.55(0.53-8.90)
	Alelo G	0.81	0.48			
	Alelo A	0.19	0.52			
miR146A	rs2910164	52	69			
	GG	23 (44.2)	29 (42.0)	0.74	GG vs GC + CC	1.14(0.40-1.92)
	GC	23 (44.2)	35 (50.7)			
	CC	6 (11.5)	5 (7.2)	0.52	GG + GC vs CC	0.65(0.18-2.43)
	Alelo G	0.66	0.67			
	Alelo C	0.34	0.33			
miR570	rs4143815	54	69			
	GG	35 (64.8)	46 (66.7)	0.82	GG vs GC + CC	1.10(0.49-2.47)

	GC	17 (31.5)	19 (27.5)			
	CC	2 (3.7)	4 (5.8)	0.32	GG + GC vs CC	2.38(0.41-13.01)
	Alelo G	0.80	0.80			
	Alelo C	0.20	0.20			
miR200B	rs9660710	55	67			
	CC	42 (76.4)	55 (82.1)	0.36	CC vs CA + AA	1.27 (0.48-3.37)
	CA	11 (20.0)	12 (17.9)			
	AA	2 (3.6)	0 (0.00)	0.27	CC + CA vs AA	0.00 (0.00)
	Alelo C	0.86	0.90			
	Alelo A	0.14	0.10			
miR26-A1	rs7372209	49	62			
	CC	19 (38.8)	33 (53.2)	0.16	CC vs CT + TT	1.78(0.79-3.99)
	CT	24 (49.0)	23 (37.1)			
	TT	6 (12.2)	6 (9.7)	0.59	CC + CT vs TT	1.42(0.39-5.14)
	Alelo C	0.63	0.71			
	Alelo T	0.37	0.28			
miR200C	rs12904	54	69			
	GG	20 (37.0)	11 (15.9)	**< 0.01**	GG vs GA + AA	**3.42(1.36-8.57)**
	GA	25 (46.3)	45 (65.2)			

	AA	9 (16.7)	13 (18.8)	0.53	GG + GA vs AA	1.39(0.50-3.89)
	Alelo G	0.60	0.48			
	Alelo A	0.30	0.52			
DROSHA	rs10035440	54	69			
	TT	36 (66.7)	40 (58.0)	0.32	TT vs CT + CC	1.50(0.66-3.39)
	CT	17 (31.5)	27 (39.1)			
	CC	1 (1.9)	2 (2.9)	0.66	TT + CT vs CC	1.73(0.14-7.12)
	Alelo T	0.82	0.77			
	Alelo C	0.18	0.23			
miR4513	rs2168518	55	69			
	GG	26 (47.3)	43 (62.3)	0.40	GG vs GA + AA	0.71(0.32-1.58)
	GA	28 (50.9)	20 (29.0)			
	AA	1 (1.8)	6 (8.7)	**0.03**	GG + AG vs AA	**7.65(0.83-70.5)**
	Alelo G	0.73	0.77			
	Alelo A	0.27	0.23			
miR219-1	rs107822	49	64			
	CC	30 (61.2)	37 (57.8)	0.77	CC vs CT + TT	0.89(0.40-1.99)
	CT	12 (24.5)	23 (35.9)			
	TT	7 (14.3)	4 (6.2)	0.18	CC + CT vs TT	2.50(0.63-9.84)

	Alelo C	0.73	0.76			
	Alelo T	0.26	0.24			
miR2053	rs10505168	52	66			
	TT	27 (51.9)	25 (37.9)	0.21	TT vs TC + CC	0.61(0.28-1.33)
	TC	19 (36.5)	29 (43.9)			
	CC	6 (11.5)	12 (18.2)	0.55	TT + CT vs CC	0.72(0.24-2.19)
	Alelo T	0.70	0.60			
	Alelo C	0.30	0.40			
miR499	rs3746444	52	69			
	GG	37 (71.2)	53 (76.8)	0.36	GG vs GA + AA	1.51(0.62-3.69)
	GA	12 (23.1)	14 (20.3)			
	AA	3 (5.8)	2 (2.9)	0.27	GG + GA vs AA	3.15(0.37-27.04)
	Alelo G	0.83	0.87			
	Alelo A	0.17	0.13			

*Sample number; *P*-values, OR and 95%CI were obtained with logistic regression adjusted for genetic ancestry.

Table S3. Genotypic distribution of the investigated polymorphisms for leprosy patients grouped in clinical form MB in comparison to the control group.

**ID**	**Genotype**	**MB (%)**	**Control (%)**	***p*-value**	**Model**	**OR (95%CI)**
miR300	rs12894467	54	68			
	TT	26 (46.4)	30 (44.1)	0.66	TT vs TC +	1.02(0.40-1.)

					CC	
	CT	21 (37.5)	26 (38.2)			
	CC	9 (16.1)	12 (17.6)	0.79	TT + TC vs CC	1.12(0.33-2.32)
	Alelo T	0.67	0.63			
	Alelo C	0.33	0.37			
miR423	rs6505162	44	59			
	AA	15 (34.1)	15 (25.4)	0.40	AA vs AC + CC	0.69(0.29-1.65)
	AC	21 (47.7)	32 (54.2)			
	CC	8 (18.2)	12 (20.3)	0.96	AA + AC vs CC	0.97(0.35-2.73)
	Alelo A	0.58	0.53			
	Alelo C	0.42	0.47			
mir604	rs2368392	40	54			
	AA	21 (52.5)	24 (44.4)	0.50	AA vs AG + GG	0.74(0.30-1.81)
	AG	18 (45.0)	29 (53.7)			
	GG	1 (2.5)	1 (1.9)	0.79	AA + AG vs GG	1.46(0.29-1.74)
	Alelo A	0.75	0.71			
	Alelo G	0.25	0.29			
pre-miR938	rs2505901	56	68			
	TT	15 (26.8)	27 (39.7)	0.07	TT vs TC + CC	0.49(0.22-1.08)

	TC	28 (50.0)	30 (44.1)			
	CC	13 (23.2)	11 (16.2)	0.21	TT + CT vs CC	0.56(0.22-1.42)
	Alelo T	0.52	0.62			
	Alelo C	0.48	0.38			
miR100	rs1834306	45	58			
	AA	20 (44.4)	25 (43.1)	0.87	AA vs AG + GG	0.94(0.41-2.12)
	AG	19 (42.2)	26 (44.8)			
	GG	6 (13.3)	7 (12.1)	0.84	AA + AG vs GG	0.88(0.26-2.98)
	Alelo A	0.66		0.65		
	Alelo G	0.34		0.35		
miR219A1	rs213210	57	69			
	AA	49 (86.0)	58 (84.1)	0.78	AA vs AG + GG	0.87(0.31-2.41)
	AG	8 (14.0)	9 (13.0)			
	GG	0 (0.00)	2 (2.9)	0.09	AA vs AG vs GG	0.00 (0.00)
	Alelo A	0.93	0.90			
	Alelo G	0.07	0.10			
DROSHA	rs639174	51	64			
	CC	16 (31.4)	30 (46.9)	**0.03**	CC vs CT + TT	**0.43(0.19-0.98)**
	CT	24 (47.1)	28 (43.8)			

	TT	11 (21.6)	6 (9.4)	0.08	CC + CT vs TT	0.39(0.13-1.18)
	Alelo C	0.55	0.69			
	Alelo T	0.45	0.31			
miR453	rs56103835	55	69			
	TT	30 (54.5)	32 (46.4)	0.47	TT vs CT + CC	0.77(0.37-1.59)
	CT	18 (32.7)	30 (43.5)			
	CC	7 (12.7)	7 (10.1)	0.64	TT + CT vs CC	1.30(0.42-1.43)
	Alelo T	0.71	0.68			
	Alelo C	0.29	0.32			
miR196A2	rs11614913	49	66			
	CC	29 (59.2)	31 (47.0)	0.15	CC vs CT + TT	1.39(0.29-2.03)
	CT	17 (34.7)	30 (45.5)			
	TT	3 (6.1)	5 (7.6)	0.71	CC + CT vs TT	1.32(0.40-4.35)
	Alelo C	0.76	0.70			
	Alelo T	0.24	0.30			
AGO	rs636832	50	66			
	GG	22 (44.0)	39 (59.1)	**0.04**	GG vs GA + AA	**0.45(0.21-0.99)**
	GA	23 (46.0)	21 (31.8)			
	AA	5 (10.0)	6 (9.1)	0.98	GG + GA vs AA	1.01(0.28-3.61)

	Alelo G	0.67	0.75			
	Alelo A	0.33	0.25			
pri-let-7a1	rs10739971	17	27			
	GG	6 (35.3)	6 (22.2)	0.23	GG vs GA + AA	2.54(0.53-12.3)
	GA	9 (52.9)	14 (51.9)			
	AA	2 (11.8)	7 (25.9)	0.13	GG + GA vs AA	3.95(0.58-27.1)
	Alelo G	0.62	0.48			
	Alelo A	0.38	0.52			
miR146A	rs2910164	56	69			
	GG	24 (42.9)	29 (42.0)	0.74	GG vs GC + CC	1.110.53-2.30)
	GC	31 (55.4)	35 (50.7)			
	CC	1 (1.8)	5 (7.2)	0.66	GG + GC vs CC	4.26(0.47-38.5)
	Alelo G	0.71	0.67			
	Alelo C	0.29	0.33			
miR570	rs4143815	57	69			
	GG	27 (47.4)	46 (66.7)	**0.03**	GG vs GC + CC	**0.45(0.21-0.96)**
	GC	21 (36.8)	19 (27.5)			
	CC	9 (15.8)	4 (5.8)	0.08	GG + GC vs CC	0.34(0.10-1.28)
	Alelo G	0.66	0.80			

	Alelo C	0.34	0.20			
miR26-A1	rs7372209	49	62			
	CC	25 (51.0)	33 (53.2)	0.85	CC vs CT + TT	1.40(0.72-2.72)
	CT	19 (38.8)	23 (37.1)			
	TT	5 (10.2)	6 (9.7)	0.66	CC + CT vs TT	1.59(0.51-4.94)
	Alelo C	0.70	0.71			
	Alelo T	0.30	0.29			
miR200C	rs12904	57	69			
	GG	16 (28.1)	11 (15.9)	0.06	GG vs GA + AA	2.28(0.93-5.58)
	GA	28 (49.1)	45 (65.2)			
	AA	13 (22.8)	13 (18.8)	0.73	GG + GA vs AA	0.86(0.35-2.11)
	Alelo G	0.52	0.48			
	Alelo A	0.48	0.52			
DROSHA	rs10035440	56	69			
	TT	44 (78.6)	40 (58.0)	**0.01**	TT vs CT + CC	**2.88(1.22-6.79)**
	CT	12 (21.4)	27 (39.1)			
	CC	0 (0.0)	2 (2.9)	0.11	TT + CT vs CC	0.00(0.00)
	Alelo T	0.89	0.77			
	Alelo C	0.11	0.24			

miR4513	rs2168518	57	69			
	GG	25 (43.9)	43 (62.3)	0.17	GG vs GA + AA	0.57(0.26-1.27)
	GA	25 (43.9)	20 (29.0)			
	AA	7 (12.3)	6 (8.7)	0.87	GG + AG vs AA	0.91(0.27-3.03)
	Alelo G	0.66	0.77			
	Alelo A	0.34	0.23			
miR219-1	rs107822	46	64			
	CC	28 (60.9)	37 (57.8)	0.59	CC vs CT + TT	0.89(0.46-1.74)
	CT	16 (34.8)	23 (35.9)			
	TT	2 (4.3)	4 (6.2)	0.54	CC + CT vs TT	1.49(0.42-5.25)
	Alelo C	0.78	0.76			
	Alelo T	0.22	0.24			
miR2053	rs10505168	53	66			
	TT	18 (34.0)	25 (37.9)	0.53	TT vs TC + CC	0.87(0.34-1.90)
	TC	26 (49.1)	29 (43.9)			
	CC	9 (17.0)	12 (18.2)	0.96	TT + CT vs CC	0.81(0.34-1.86)
	Alelo T	0.58	0.59			
	Alelo C	0.42	0.41			
miR499	rs3746444	57	69			

	GG	42 (73.7)	53 (76.8)	0.80	GG vs GA + AA	1.20(0.57-2.50)
	GA	14 (24.6)	14 (20.3)			
	AA	1 (1.8)	2 (2.9)	0.84	GG + GA vs AA	1.62(0.24-10.69)
	Alelo G	0.86	0.87			
	Alelo A	0.14	0.13			

*Sample number; *P*-values, OR and 95%CI were obtained with logistic regression adjusted for genetic ancestry.

Table S4. Genotypic distribution of the investigated polymorphisms between leprosy patients grouped according clinical form MB and PB.

ID	Genotype	PB (%)	MB (%)	*p*-value	Model	OR (95%CI)
miR300	rs12894467	55	53			
	TT	18 (32.7)	25 (47.2)	0.09	TT vs CT + CC	0.48(0.20-1.14)
	CT	24 (43.6)	20 (37.7)			
	CC	13 (23.6)	8 (15.1)	0.08	TT + CT vs CC	0.39(0.13-1.16)
	Alelo T	0.54	0.66			
	Alelo C	0.46	0.34			
miR423	rs6505162	49	41			
	AA	12 (24.5)	15 (36.6)	0.46	AA vs AC + CC	0.69(0.26-1.87)
	AC	26 (53.1)	18 (43.9)			
	CC	11 (22.4)	8 (19.5)	0.99	AA + AC vs CC	1.00(0.33-3.08)
	Alelo A	0.51	0.59			

	Alelo C	0.49	0.41			
mir604	rs2368392	43	37			
	AA	21 (48.8)	19 (51.4)	0.52	AA vs AG + GG	0.73(0.28-1.94)
	AG	22 (51.2)	17 (45.9)			
	GG	0 (0.0)	1 (2.7)	0.14	AA + AG vs GG	0.00(0.00)
	Alelo A	0.74	0.74			
	Alelo G	0.26	0.26			
pre-miR938	rs2505901	53	53			
	TT	17 (32.1)	12 (22.6)	0.18	TT vs CT + CC	1.90(0.74-4.90)
	CT	25 (47.2)	27 (50.9)			
	CC	11 (20.8)	14 (26.4)	0.07	TT + CT vs CC	2.56(0.89-7.38)
	Alelo T	0.56	0.48			
	Alelo C	0.44	0.52			
miR100	rs1834306	48	42			
	AA	24 (50.0)	18 (42.9)	0.45	AA vs AG + GG	1.41(0.57-3.45)
	AG	17 (35.4)	19 (45.2)			
	GG	7 (14.6)	5 (11.9)	0.65	AA + AG vs GG	0.74(0.20-2.76)
	Alelo A	0.68	0.65			
	Alelo G	0.32	0.35			
DROSHA	rs639174	50	48			
	CC	16 (32.0)	14 (29.2)	0.73	CC vs CT + TT	0.85(0.32-2.22)
	CT	27 (54.0)	23 (47.9)			

	TT	7 (14.0)	11 (22.9)	0.85	CC + CT vs TT	1.11(0.35-3.51)
	Alelo C	0.59	0.53			
	Alelo T	0.41	0.47			
miR453	rs56103835	54	52			
	TT	30 (55.6)	28 (53.8)	0.69	TT vs CT + CC	0.84(0.36-1.96)
	CT	20 (37.0)	17 (32.7)			
	CC	4 (7.4)	7 (13.5)	0.27	TT + CT vs CC	2.16(0.53-8.86)
	Alelo T	0.74	0.70			
	Alelo C	0.26	0.30			
miR196A2	rs11614913	52	46			
	CC	32 (61.5)	27 (58.7)	0.59	CC vs CT + TT	1.27(0.52-3.08)
	CT	14 (26.9)	16 (34.8)			
	TT	6 (11.5)	3 (6.5)	0.73	CC + CT vs TT	1.27(0.52-3.08)
	Alelo C	0.75	0.76			
	Alelo T	0.25	0.24			
AGO	rs636832	52	47			
	GG	24 (46.2)	20 (42.6)	0.52	GG vs GA + AA	1.33(0.55-3.18)
	GA	20 (38.5)	22 (46.8)			
	AA	8 (15.4)	5 (10.6)	0.97	GG + GA vs AA	1.02(0.27-3.80)
	Alelo G	0.65	0.66			
	Alelo A	0.35	0.34			
pri-let-7a1	rs10739971	13	16			

	GG	9 (69.2)	6 (37.5)	**0.01**	GG vs GA + AA	**5.21(1.59-7.86)**
	GA	3 (23.1)	9 (56.2)			
	AA	1 (7.7)	1 (6.2)	0.55	GG + GA vs AA	2.68(0.11-6.46)
	Alelo G	0.81	0.65			
	Alelo A	0.19	0.36			
miR146A	rs2910164	52	53			
	GG	23 (44.2)	24 (45.3)	0.25	GG vs GC + CC	0.60(0.24-1.46)
	GC	23 (44.2)	28 (52.8)			
	CC	6 (11.5)	1 (1.9)	**0.04**	GG + GC vs CC	**0.14(0.01-1.36)**
	Alelo G	0.66	0.72			
	Alelo C	0.34	0.28			
miR570	rs4143815	54	54			
	GG	35 (64.8)	26 (48.1)	0.17	GG vs GC + CC	1.79(0.77-4.17)
	GC	17 (31.5)	20 (37.0)			
	CC	2 (3.7)	8 (14.8)	0.28	GG + GC vs CC	2.38(0.44-12.79)
	Alelo G	0.80	0.66			
	Alelo C	0.20	0.34			
miR200B	rs9660710	55	45			
	CC	42 (76.4)	35 (77.8)	0.98	CC vs CA + AA	1.01 (0.35-2.91)
	CA	11 (20.0)	10 (22.2)			
	AA	2 (3.6)	0 (0.0)	0.10	CC + CA vs AA	0.00 (0.00)

	Alelo C	0.86	0.88			
	Alelo A	0.14	0.11			
miR26-A1	rs7372209	49	46			
	CC	19 (38.8)	23 (50.0)	0.10	CC vs CT + TT	0.47(0.19-1.18)
	CT	24 (49.0)	18 (39.1)			
	TT	6 (12.2)	5 (10.9)	0.52	CC + CT vs TT	0.64(0.17-2.50)
	Alelo C	0.63	0.70			
	Alelo T	0.37	0.30			
miR200C	rs12904	54	54			
	GG	20 (37.0)	15 (27.8)	0.26	GG vs AG + AA	1.66(0.68-4.06)
	AG	25 (46.3)	26 (48.1)			
	AA	9 (16.7)	13 (24.1)	0.41	GG + AG vs AA	1.54(0.54-4.37)
	Alelo G	0.60	0.52			
	Alelo A	0.40	0.48			
DROSHA	rs10035440	54	53			
	TT	36 (66.7)	41 (77.4)	0.63	TT vs CT + CC	0.80(0.31-2.05)
	CT	17 (31.5)	12 (22.60			
	CC	1 (1.9)	0 (0.0)	0.47	TT + CT vs CC	0.00(0.00)
	Alelo T	0.82	0.89			
	Alelo C	0.17	0.11			
miR4513	rs2168518	55	54			
	GG	26 (47.3)	23 (42.6)	0.70	GG vs GA + AA	1.56(0.80-3.04)

	GA	28 (50.9)	25 (40.3)			
	AA	1 (1.8)	6 (11.1)	0.09	GG + GA vs AA	5.37(0.56-9.89)
	Alelo G	0.73	0.66			
	Alelo A	0.27	0.34			
miR219-1	rs107822	49	43			
	CC	30 (61.2)	26 (60.5)	0.83	CC vs CT + TT	0.91(0.36-2.30)
	CT	12 (24.5)	15 (34.9)			
	TT	7 (14.3)	2 (4.7)	0.07	CC + CT vs TT	0.23(0.04-1.31)
	Alelo C	0.73	0.78			
	Alelo T	0.27	0.22			
miR2053	rs10505168	52	50			
	TT	27(51.9)	17 (34.0)	0.06	TT vs TC + CC	2.31(0.95-5.61)
	TC	19 (36.5)	24 (48.0)			
	CC	6 (11.5)	9 (18.0)	0.51	TT + TC vs CC	1.50(0.45-5.06)
	Alelo T	0.70	0.58			
	Alelo C	0.30	0.42			
miR499	rs3746444	52	54			
	GG	37 (71.2)	40 (74.1)	0.97	GG vs GA + AA	1.01(0.39-2.61)
	GA	12 (23.1)	13 (24.1)			
	AA	3 (5.8)	1 (1.9)	0.25	GG + GA vs AA	0.25(0.02-3.05)
	Alelo G	0.82	0.86			
	Alelo A	0.17	0.14			

*Sample number; *P*-values, OR and 95%CI were obtained with logistic regression adjusted for age and sex.

Table S5. Characterization of the polymorphisms studied.

Gene	ID	Alleles	Function	MAF*
miR20b/miR-17-5P	rs3660	C>G	3 Prime UTR Variant	48%
miR300	rs12894467	C>T	Non-Coding Transcript exon variant	39%
miR423	rs6505162	C>A	Non-Coding Transcript variant	50%
mir604	rs2368392	G>A	Non-Coding Transcript Variant	32%
pre-miR938	rs2505901	C>T	Intron Variant	40%
miR605	rs2043556	T>C	Non Coding Transcript Variant	25%
miR100	rs1834306	A>G	Intron Variant	45%
miR219A1	rs213210	A>G	Regulatory Region Variant	17%
DROSHA	rs639174	C>T	Intron Variant	46%
miR453	rs56103835	T> C	Non-Coding Transcript Exon Variant	30%
miR196A2	rs11614913	C>T	Non-Coding Transcript Exon Variant	33%
AGO1	rs636832	A>G	Intron Variant	36%
pri-let-7a-1	rs10739971	G>A	Intron Variant	26%
miR146A	rs2910164	C>G	Mature miRNA Variant	29%
miR570	rs4143815	G>C	3 prime UTR ariant	28%
miR200B	rs9660710	A>C	Regulatory Region Variant	10%
miR26-A1	rs7372209	C>T	Intron Variant	20%
miR200C	rs12904	G> A	3 Prime UTR Variant	49%
DROSHA	rs3805500	G>A	Intron Variant	49%
DROSHA	rs10035440	T>C	Intron Variant	15%
miR4513	rs2168518	G> A	Non-Coding Transcript Exon Variant	24%
miR219-1	rs107822	C>T	TF Binding Site	37%
miR149	rs2292832	T>C	Intron Variant	38%
miR2053	rs10505168	C>T	Non Coding Transcript Variant	39%

miR499	rs3746444	A>G	Mature miRNA Variant	18%

*Minor Allele Frequency according 1000 Genomes Project.
